# Metastasis of small cell lung cancer to bilateral extraocular muscles: a case report

**DOI:** 10.1186/s13256-024-04525-z

**Published:** 2024-05-02

**Authors:** João Ponces Ramalhão, Beatriz Costa Vieira, Diogo Rodrigues, Miguel Gonçalves Afonso, João Gouveia, Pedro Manuel Baptista, Maria Araújo

**Affiliations:** 1 Serviço de Oftalmologia, Centro Hospitalar Universitário do Porto Largo Professor Abel Salazar, 4099-001 Porto, Portugal; 2 Serviço de Medicina Interna, Hospital Dr. Nélio Mendonça, Avenida Luís de Camões 6180, 9000-177 Funchal, Portugal

**Keywords:** Orbital metastasis, Small cell lung cancer, Proptosis

## Abstract

**Background:**

Orbital metastasis is a possible complication of small cell lung cancer and a pattern of bilateral invasion of the extraocular muscles has rarely been reported in literature.

**Case presentation:**

A 46-year-old white male with a past medical history of smoking and stage IV small cell lung carcinoma presented with loss of vision and pain in the left eye. Examination revealed bilateral proptosis and left afferent pupillary defect, and visual acuity was hand motion on the left eye and 4/10 on the right eye. An orbital computed tomography scan showed a compression of the left optic nerve between the extraocular muscles at the apex, and a lateral canthotomy was performed for a new-onset compressive optic neuropathy, with residual visual improvement. There was also significant enlargement of the extraocular muscles in the right orbit. The patient was maintained in palliative treatment with both chemotherapy and local medical and surgical (amniotic membrane cover for exposure keratopathy) ophthalmological treatments until he eventually died 5 months after.

**Conclusion:**

Bilateral metastasis to the extraocular muscles is a very rare manifestation of small cell lung cancer and the palliative treatment in these cases is challenging.

## Background

Orbital metastasis is a relatively uncommon entity, but they can be the first sign of advanced neoplasms. These metastases typically affect one eye, leading to signs and symptoms such as proptosis, pain, vision loss, or double vision [1]. Unfortunately, the prognosis for patients with orbital metastasis is often poor. However, there are some treatment options available to help alleviate symptoms and improve the patient’s quality of life, such as systemic chemotherapy and targeted radiation therapy.

We present a case of bilateral orbital metastasis that arose from an advanced stage IV small cell lung cancer (SCLC). In this case, the metastasis had infiltrated the extraocular muscles, which is an uncommon presentation and has only been reported a few times in the medical literature [2]. Despite the rarity of this condition, it is important to be aware of the possibility of bilateral orbital metastasis and to consider them in the differential diagnosis when evaluating patients with advanced cancer. While the prognosis for these patients may be poor, prompt diagnosis and appropriate palliative care can help to improve their comfort and overall quality of life.

## Case presentation

A 46 year-old white male with stage IV small cell lung carcinoma presented to the ophthalmological emergency department. In addition to his oncological situation, past medical history disclosed smoking for 10 years and asthma since childhood. He was diagnosed 1 year earlier after complaining of dorsal and thoracic pain associated with dyspnea, anorexia, and loss of weight. A thoracic, abdominal, and pelvic computed tomography (CT) at that time revealed multiple vertebral and iliac hyperdense areas suggestive of bone metastasis. Laboratory analyses at that time revealed normal protein levels with no monoclonal spikes on electrophoresis, a normal total prostate specific antigen (0.7 ng/mL; *N* < 4 ng/mL), moderate C-reactive protein (1.2 mg/dL, *N* < 1.0 mg/dL), and no microalbuminuria. Beta-Human chorionic gonadotropin (B-HCG), Alpha Fetoprotein (AFP) and Lactate dehydrogenase (LDH) were normal. CT angiography revealed pleural and lung nodules as well as enlarged mediastinal lymph nodes. A transthoracic aspirative biopsy of the mediastinal adenopathies was performed and histological analysis revealed small round and blue cells with trabecular and nest-like architecture with many mitotic figures. Immunohistochemistry showed reactivity to CAM5.2, chromogranin, synaptophysin, and CD56 with negative CD45, CK7, CK20, and TTF-1. The proliferative index was high (Ki-67 more than 20%). All these aspects pointed to a metastasis of a neuroendocrine tumor with an assumed primary origin in the lung, and the patient initiated palliative treatment with systemic chemotherapy (cisplatin and etoposide). Positron emission tomography-fluorodeoxyglucose (PET-FDG) scan revealed a mediastinal hypermetabolic mass (compatible with ganglionar metastization), two bone lesions at the sacrum and humerus, and an avid superior right lobe lung nodule suggestive of being the primary lesion. No activity was noted at that time at the extraocular muscles. Due to the patient’s frailty, he interrupted previous chemotherapy after six cycles and received second-line palliative treatment with topotecan.

**Fig. 1:**
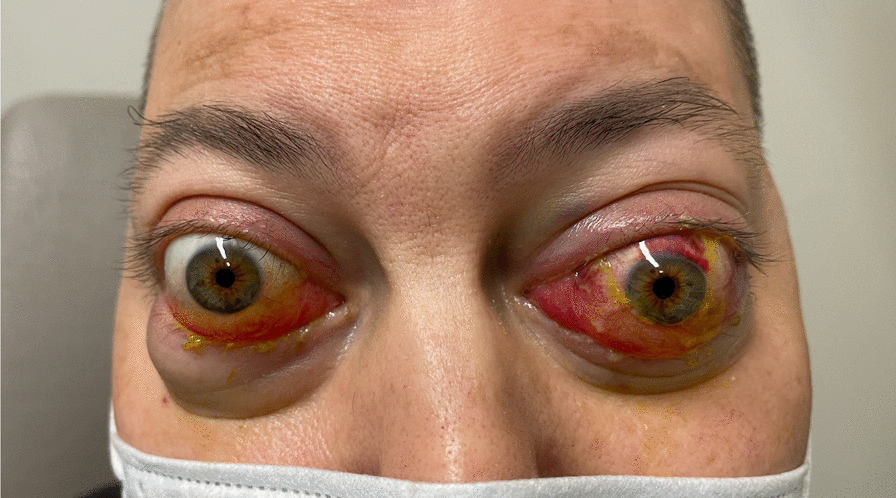
Bilateral orbital proptosis in our patient

During this period, he complained of loss of vision and ocular pain. He was observed by our ophthalmology department. Our evaluation revealed proptosis in both eyes (OU), limitation of ocular movements OU, lagophthalmos OU [with absent Bell phenomenon in the left eye (LE)], and a new-onset LE relative afferent pupillary defect (APD) (Fig. 1). His visual acuity was 4/10 in the right eye (RE) and hand-motion in the LE (6/10 and 3/10, respectively, 1 month earlier). Biomicroscopy revealed an exposure keratopathy OU. On fundus examination, RE had choroidal folds and normal optic disc and LE had both choroidal folds and optic disc edema.

**Fig. 2:**
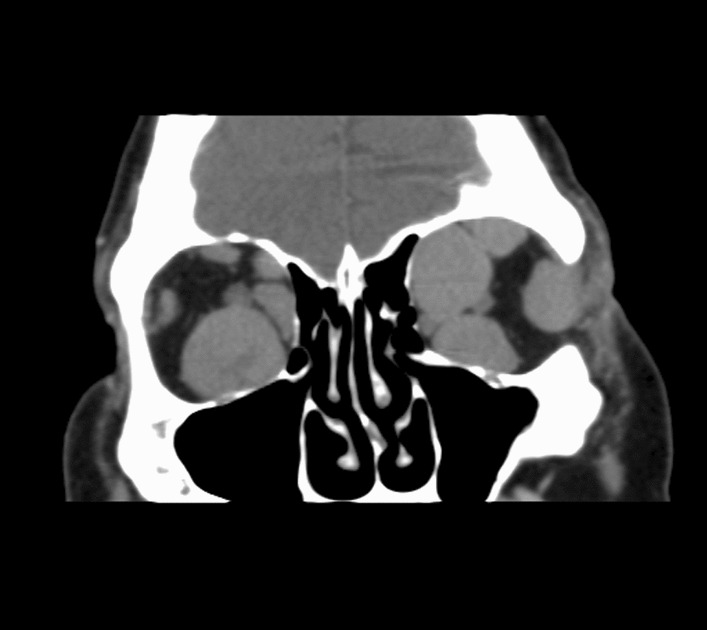
Orbital computed tomography (axial) revealing extraocular muscle infiltration

**Fig. 3:**
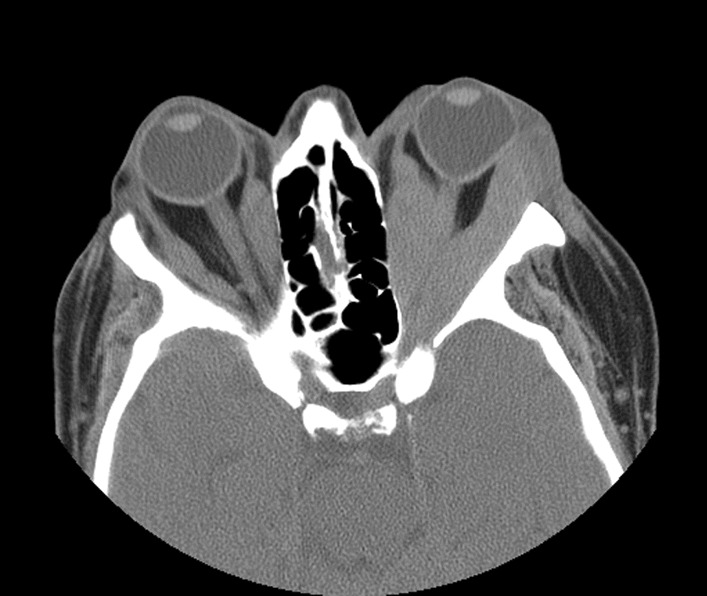
Orbital computed tomography (coronal)

Orbital computed tomography (CT) scan revealed bilateral infiltration of all extraocular muscles (Figs. 2 and 3), with orbital compression on the left orbit leading to compression of the optic nerve by the enlarged muscles at the level of the apex (previous CT scans of this patient showed no space conflict at the apex of both orbits).

After multidisciplinary discussion with oncology and maxillofacial surgery, a decision to decompress the left orbit with a lateral canthotomy due to a compressive optic neuropathy was made, in accordance with the patient.

Although visual acuity of the left eye improved slightly (to 1/10), there was progression of the proptosis and worsening of the exposure keratopathy, and an intensive lubrication and amniotic membrane cover was performed on the LE. Incisional biopsy of extraocular muscle metastases was not performed to reduce the patient burden as it would not alter the treatment course.

The patient remained under palliative chemotherapy and eventually died 5 months after presentation.

## Discussion and conclusions

Orbital metastasis is rare. The most frequently involved sites of origin include breast, lung, and prostate carcinoma [1]. Curiously, hepatocellular and gastric cancer orbital metastases correspond to the most frequent etiology in Japanese patients [3, 4]. Extraocular muscle metastases represent 9% of orbital metastasis. Isolated extraocular muscle metastases have been reported especially in cases of carcinoid tumor and prostate cancer [5, 6].

Bilateral metastization is much less common than unilateral and breast cancer; neuroblastoma and lymphoma are the most common primaries in such instances [7]. SCLC is the most aggressive type of lung cancer and its tendency for distance metastization is known [8]. The most frequent site of metastasis is the brain, accounting for more than two-thirds of the cases [9]. Orbital metastasis occurs in up to 12% of patients with lung cancer and SCLC orbital metastasis tend to present as a soft tissue mass with adjacent bony destruction of the orbit [10].

Clinical presentation of orbital metastasis may vary depending on the affected structure. Common features such as exophthalmos and pain may be accompanied by ocular motility defects and diplopia or APD and loss of vision when there is involvement of the extraocular muscles and optic nerve [11].

Diagnosis of orbital metastasis implies pathological confirmation with either an open biopsy or fine-needle aspiration biopsy (FNAB). Nevertheless, posterior orbital biopsies carry several risks, such as uncontrollable bleeding, infection of orbital tissues, and subsequent vision loss. If a patient is known to have a metastatic tumor, with lesions most likely to resemble metastasis on orbital CT and magnetic resonance imaging (MRI), an assumption of orbital spreading of malignant disease can be made [12].

Although there are treatment options in patients with orbital metastases to control local disease, the prognosis in these patients remains limited to the progression of systemic disease. Radiotherapy regimens of the orbit include palliative fractioned radiation at doses of 30–40 Grays (Gy). In addition, systemic chemotherapy may aid in the control of orbital disease, with the most common treatment regimen in SCLC being cisplatin plus etoposide, as in our case. Despite these interventions, mean survival time after orbital metastasis is 20 months, and even lower (4 months) in patients in whom the primary tumor is lung cancer [4].

Few cases of orbital metastases from SCLC have been described in literature [3, 13, 14]. Tezcan *et al*. described a case of a bilateral SCLC metastasis in a 46-year-old male patient. This patient presented with two orbital solid masses invading on the right side the optic nerve and on the left side the lateral wall. Infiltration of the extraocular muscles was not a feature in this case. Although a pathological confirmation of the orbital metastases was not made, palliative orbital radiotherapy of 30 Gy/10 days was performed [4]. Crisostomo *et al*. described a case of a 56-year-old-male patient with bilateral extraocular metastatic disease affecting the extraocular muscles. They noted a more advanced disease on the left orbit, as in our case, which has been theorized as a consequence of the anatomic configuration of the arterial supply to the orbit from the carotid. An incisional biopsy of the extraocular muscle was performed in this case to confirm diagnosis [2]. Other cases described in the literature of isolated extraocular muscle involvement of the orbit are listed in Table 1 [2, 15–17].

**Table 1:** Reported cases of EOM in SCLC in literature

Author, date	Laterality	Muscles involved	Biopsy	Type	Other metastasis
Divine and Anderson *et al*., 1982	Unilateral	1	Yes	SCLC	Yes
Benson, Parsons, and Rennie, 1993	Bilateral	2 (lateral rectus LE; medial rectus RE)	Yes	SCLC	Yes
Kim *et al*., 2017	Unilateral	1 (medial rectus RE)	Yes	SCLC	No
Crisostomo *et al*., 2019	Bilateral	2 (medial rectus LE, lateral rectus RE)	Yes	SCLC	Yes

*EOM* extraocular muscles; *SCLC* small cell lung cancer; *LE* left eye; *RE* right eye

Furthermore, management of proptosis sequelae and exposure keratopathy may be important in these patients to reduce pain and complications. Procedures such as amniotic membrane cover, as in our patient, may palliate pain and improve comfort.

## Data Availability

All data are available at our institutional records and may be accessed upon request. All data generated or analyzed during this study are included in this article. Further inquiries can be directed to the corresponding author.
